# Supplementary material for: Patterns of Intron Gain and Loss in Fungi
Source: PLoS Biol. 2004 Nov 30;2(12):e422. doi: 10.1371/journal.pbio.0020422 (PMC532390; doi:10.1371/journal.pbio.0020422)
Supplement: Table S1 — Also available at http://genes.mit.edu/NielsenEtAl/. (4.3 MB ZIP). [file pbio.0020422.st001.zip › NielsenEtAl/html/396.html]

AN5606.1.NCU03463.1.MG03947.1.FG00818.1


```
 CLUSTAL W (1.82) Multiple Sequence Alignments - Introns Inserted


Sequence 1: NCU03463.1	856 aa
Sequence 2: MG03947.1	850 aa
Sequence 3: FG00818.1	857 aa
Sequence 4: AN5606.1	852 aa
Alignment Length: 872 aa
Number Identitical Residues: 504 aa
Alignment Score (without introns) 23754


MG03947.1 	MAHTGDTPFRSAEMSMVQLYISNEIGREVVNALGEVGLVQFRD0LNGDLSAFQRAFTQEI
NCU03463.1	MAPKQDTPFRSADMSMVQLYISNEIGREVCNALGELGLVHFRD0LNSELSAFQRAFTQDI
FG00818.1 	MAPKPDTPFRSADMSMVQLYVSNEIGREVVTALGELGLCQFRD0LNENVSAFQRTFTQEI
AN5606.1  	MAPK-DTFFRSSDMSLTQLYIANEIGREVVSALGELGQVQFRD0LNPETNAFQKTFTKEI
          	** . ** ***::**:.***::******* .****:*  :*** ** : .***::**::*

MG03947.1 	RRLDNVERQLR1YFHAQMEKAGIPLRKLDLDVESLAPPSTSEIDELADRSQSLEQRISQL
NCU03463.1	RRLDNVERQLR1YFHSQMEKAGIPLRKFDPDVDILTPPTTTEIDELAERAQTLEQRVSSL
FG00818.1 	RRLDNVERQLR1YFYAQMDKIGIPLRKLDLDVERLASPSTSEIDELAERSQKLEQRVSAL
AN5606.1  	RRLDNVERQLR1YFHAQMDKAGIQMRPSSEFSDTLAAPLASEIDELAERSESLEQRIASL
          	*********** **::**:* ** :*  .   : *:.* ::******:*::.****:: *

MG03947.1 	NDSYETLKKREVELTEWRWVLREAGGFFDRAHGNVEEIRASTEDDDAPLLQDVEQHNQG-
NCU03463.1	NESYETLKKREVELTEWRWVLREAGGFFDRAHGNVEEIRASTDNDDAPLLQDVEQHNTA-
FG00818.1 	NESYETLKKREGDLTEWRWVLREAGSFFDRAHGNVEEIRASTDNDDAPLLSDIENNQSG-
AN5606.1  	NDSYETLKKREVELTEWRWVLREAGGFFDRAHTHTEEIRQSFDNDEAPLLRDVEQQNHRG
          	*:********* :************.****** :.**** * ::*:**** *:*:::  .

MG03947.1 	----GDVERSFSGMNIGFVAGVIARERVAAFERILWRTLRGNLYMNQSEIPEPLVDPSNN
NCU03463.1	----ADVERSFSGMNIGFVAGVIGRDRVDAFERILWRTLRGNLYMNQAEIPEPLIDPTIN
FG00818.1 	----ADADRSFSGMNIGFVAGVIARDRVASFERILWRTLRGNLYMNQSEIPEPLIDPTNN
AN5606.1  	ANGDAQGQQSFLELNIGFVSGVIPRDRIGAFERILWRTLRGNLYMNQAEIPDPIVDPTTN
          	:....: ::**  :*****:*** *:*: :*****************:***:*::**: *

MG03947.1 	EPIQKNVFVIFAHGKEILAKVRKISESMGAEVYNVDENSDLRRDQVFEVNARLNDVQNVL
NCU03463.1	EPVLKNVFVIFAHGKEILAKIRRISESMGAEVYNVDEHSDLRRDQVHEVNARLEDVQNVL
FG00818.1 	EAINKNVFVIFAHGKEILNKIRKISESMGADVYNVDENSDLRRDQIHEVNNRLEDVQNVL
AN5606.1  	EETQKMVFVIFAHGKNIIAKIRKISESLGASLYSVDENSELRRDQIHEVNTRLSDVNNVL
          	*   * *********:*: *:*:****:**.:*.***:*:*****:.*** **.**:***

MG03947.1 	KNTQQTLDAELTQISQSLAAWMVLINKEKAVYNTLNLFSYDRARRTLIAEGWCPKNDLPL
NCU03463.1	RNTQQTLEAELAQISQSLSAWMITISKEKAVYNTLNLFSYDRARRTLIAEGWCPTNDLPL
FG00818.1 	QNTQATLQAELNQISQSLSAWMVLVAKEKAVYNALNNFSYDSARRTLIAEAWVPTNDLPL
AN5606.1  	RNTKNTLDAELSQIARSLAAWMIIVKKEKAVYDTLNKCSYDQARKTLIAEAWCPTNSLSL
          	:**: **:*** **::**:***: : ******::**  *** **:*****.* *.*.*.*

MG03947.1 	IRTTLQDVTNRAGLSVPSIINEIRTNKKPPTYLKTNKFTEAFQTIVNAYGTATYQEVNPA
NCU03463.1	IRSTLQDVNNRAGLSVPSIINEIRTNKTPPTYLKTNKFTEAFQTIVNAYGTATYQEVNPA
FG00818.1 	IRTTLQEVTNRAGLSVPSIINKIQSNKTPPTYLKTNKFTEGFQTIVNAYGTATYQEVNPA
AN5606.1  	IKSTLQDVNDRAGLSVPSIVNQIRTNKTPPTYVRTNKFTEAFQTIVDAYGISKYSEVNPG
          	*::***:*.:*********:*:*::**.****::******.*****:*** :.*.****.

MG03947.1 	LPVIVTFPFLFAVMFGDLGHALIMLCAALAMIYWEKPLKKVSF-ELFAMVYYGRYIALVM
NCU03463.1	IPVIVTFPFLFAVMFGDFGHALIMLCAALAMIYWEKPLKKVTF-ELFAMVFYGRYIVLVM
FG00818.1 	MPVFVTFPFLFAVMFGDFGHAIIMLSAALAMIYWEKSLKKVSF-ELFAMIFYGRYIALVM
AN5606.1  	LYTVVTFPFLFAVMFGDFGHGFLMALAAAAMIFWERQLSKTKLDELTYMAFYGRYIMLMM
          	: ..*************:**.::*  ** ***:**: *.*..:.**  * :***** *:*

MG03947.1 	AVFSVFTGLVYNDIFSKSMTFWDSAWEWDVPADYKDFTTVTAKLKG--DYRYPFGLDWMW
NCU03463.1	AVFSVYTGLIYNDVFSKSMTLFDSQWKWVVPENFKEGMTVKAVLREPNGYRYPFGLDWRW
FG00818.1 	AVFSVFTGLVYNDVFSMSMTLFPSAWEWKKPDNYSNTTSIIATLNE-EGYRYPFGLDYAW
AN5606.1  	GIFSMYTGLIYNDIFSKSFTVFSSSWKW--PDNIEQGQSVEASLKG--SYRFPFGLDWNW
          	.:**::***:***:** *:*.: * *:*  * : .:  :: * *.   .**:*****: *

MG03947.1 	HGTENDLLFSNSYKMKMSIILGWAHMTYSLCFSYINARHFKKPIDIWGNFVPGMIFFQSI
NCU03463.1	HGTENELLFINSYKMKMAIILGWAHMTYSLCFSYINARHFKRPIDIWGNFVPGMIFFQSI
FG00818.1 	HGSENDLLFSNSLKMKMSIILGWAHMTYSLCFAYINARHFKKPIDIWGNFIPGMIFFQSI
AN5606.1  	HEAENSLLFTNSLKMKMSIILGWAHMTYALILQYVNARHFKSKVDIIGNFIPGIIFFQSI
          	* :**.*** ** ****:**********:* : *:******  :** ***:**:******

MG03947.1 	FGYLVICIIYKWTIDWSKADTAPPGLLNMLIYMFLQPGTID--VQLYPGQKPVQIFLLLL
NCU03463.1	FGYLVLCIIYKWSVDWFGTGRQPPGLLNMLIYMFLQPGTLDGGVELYPGQATVQVILLLL
FG00818.1 	FGYLVLCIIYKWSVDWNGLKLNPPGLLNMLIYMFLQPGTIPEGQELYSGQGFVQVVLLLL
AN5606.1  	FGYLVLTIIYKWSVDWPARNQSPPGLLNMLIFMFLSPGNVE--EELYPGQGGVQLCLLLL
          	*****: *****::**      *********:***.**.:    :**.**  **: ****

MG03947.1 	AFAQVPILLFLKPFYLRWEHNRARGQGYRGIGERSHVSAFDDDNDDGNGHAMNGGRGNSL
NCU03463.1	AVIQVPILLFLKPFYLRWENNRARAKGYRGIGERSRVSALDEDDEE---DPSNG------
FG00818.1 	AFIQVPILLCLKPFYLRWENNRARAKGYRSIGETSRVSALDGDEDEPNGHGNSFD-----
AN5606.1  	AVAQVPIMLFFKPFYLRREHNRARALGYRGLGEQSRVSALDEDGDLDGPRQSTAS-----
          	*. ****:* :****** *:****. ***.:** *:***:* * :  .    . .     

MG03947.1 	DSDSGAAMITQDIH-DEEHEEFEFGEVMIHQVIHTI-~---------A1SYLRLWALSLA
NCU03463.1	DDYEGAAMLTHDEHGDGEHEEFEFGEVMIHQVIHTIE1FCLNSVSHTA~SYLRLWALSLA
FG00818.1 	EDGEGVAMISQNID--EEHEEFEFSEVMIHQVIHTIE1FCLNCVSHTA~SYLRLWALSLA
AN5606.1  	-DGEGVAMIAQDLE--EEHEEFDFSEIMIHQVIHTIE~FCLNCISHTA~SYLRLWALSLA
          	 . .*.**:::: .   *****:*.*:*********.  . .. : :* ***********

MG03947.1 	HQQLSVVLWSMTIGQTFAFT-GILGGIAVFLGFAVFFVLS~VIILIIMEGVSAM~LHS1L
NCU03463.1	HQQLSAVLWSMTMAKALESK-GLGGAIFLVVAFAMFFVLS~VIILIIMEGVSAM0LHS~L
FG00818.1 	HQQLSIVLWSMTLGPALKTP-GVMGVIMIVVCFTMWFFLT1IAILVCMEGTSAM~LHS~L
AN5606.1  	HQQLSIVLWDMTLGTAFDQEDGTIRTIMIIVTFYMWFTLT~IAILCVMEGTSAM~LHS~L
          	***** ***.**:. ::   .*    * :.: * ::* *: : **  ***.*** *** *

MG03947.1 	RLAWVESFSKFAEFAGWPFQPFSFTQQIEESEELKEFLG
NCU03463.1	RLAWVESFSKFAEFGGWPFTPFSFKQQLEESEELKEYIG
FG00818.1 	RLAWVESFSKFAEFAGWPFAPFSFNTLLEESEELKDYLG
AN5606.1  	RLHWVEAMSKHFMGDGIPFAPFSFKTLLEEDPVD-----
          	** ***::**.    * ** ****.  :**.
```
